# Unmasking inflammation in juvenile dermatomyositis: myokine profiles of patients and bioengineered human muscle

**DOI:** 10.3389/fimmu.2025.1694717

**Published:** 2025-12-18

**Authors:** Lauren T. Covert, George A. Truskey, Sara Kandil, Jessica Neely, Jessica L. Turnier, Kaveh Ardalan, Jeffrey A. Dvergsten

**Affiliations:** 1 Department of Pediatrics, Duke University School of Medicine, Durham, NC, United States; 2 Department of Biomedical Engineering, Duke University, Durham, NC, United States; 3 Department of Pediatrics, University of California, San Francisco, CA, United States; 4 Department of Pediatrics, University of Michigan, Ann Arbor, MI, United States

**Keywords:** dermatomyositis, idiopathic immune myopathy, myokine, cytokine, interferon, bioengineered tissue, autoimmunity, pediatric rheumatology

## Abstract

**Introduction:**

Juvenile dermatomyositis (JDM), a rare autoimmune disease characterized by a type I interferon (IFN) gene signature and muscle weakness, lacks robust biomarkers and disease models. Myokines—muscle-derived cytokines—are potential biomarkers and therapeutic targets that may clarify muscle’s role in JDM. We characterized myokine profiles in treatment-naïve JDM patients and compared them to an IFN-stimulated human tissue-engineered muscle model (myobundles) to identify biomarkers and validate the model.

**Methods:**

Myobundles from four healthy pediatric donors were treated with IFNα, IFNβ, or IFN-stimulant poly(I:C). Sera from treatment-naïve JDM patients (n=21) and controls (n=9) were analyzed. A myokine panel (e.g., IL-6, IL-8, IL-17A, IL-18, CXCL9, CXCL10, TNFα, RANTES, IFNα-2a, IFNβ) was quantified in myobundle media and patient sera, with gene expression assessed by RNA sequencing in myobundles and JDM muscle biopsies. Serum myokines were correlated with Childhood Myositis Assessment Score (CMAS), and myobundle profiles were compared to patient signatures.

**Results:**

Poly(I:C) triggered the strongest myokine response in myobundles, significantly increasing IL-6, IL-8, IL-15, IL-18, CXCL9, CXCL10, RANTES, and IFNβ. IFNα treatment increased TNFα, while IFNβ upregulated IL-15. JDM sera also showed elevations in IL-6, IL-15, IL-18, CXCL9, CXCL10, and IFNβ, with additional increases in IL-17 and IFNα (p_adj_ = 0.0001–0.03). CXCL9, CXCL10, and IL-6 were significant independent predictors of CMAS, unlike conventional muscle enzymes. RNA sequencing confirmed elevated CXCL9 and CXCL10 expression in both IFN-treated myobundles and JDM biopsies. The myokine signature of IFNα-treated myobundles most closely reflected the JDM patient profile.

**Conclusion:**

JDM patients have a pro-inflammatory myokine profile in blood and muscle that can be recapitulated in IFN-stimulated myobundles. CXCL9 and CXCL10 are promising biomarkers, as are IL-6, IL-15, and IL-18, for JDM muscle activity. Our findings validate the myobundle model as a platform for studying JDM and support muscle as a key source of pathologic inflammation.

## 1 Introduction

Juvenile dermatomyositis (JDM) is a rare autoimmune disease that typically causes proximal muscle weakness and inflammation in children. Pathophysiology of JDM is characterized by a combination of vasculopathy and dysregulation of both the innate and adaptive immune system. While pathogenesis is not clearly understood, it is well established that the muscle, skin, and blood of patients with JDM have an upregulated type I interferon (IFN) gene signature (1, 2). Although myositis-specific and myositis-associated antibodies have advanced prognostication and monitoring for associated phenotypic complications, biomarkers for the diagnosis and monitoring of disease activity remain limited in the clinical setting. Typical surrogate markers of muscle damage, such as creatine kinase, aldolase, and lactate dehydrogenase, correlate poorly with disease activity, especially in longstanding disease. Furthermore, despite known IFN gene signature, accurate quantification of type I interferons in patients is difficult with contemporary commercial assays (3). Meeting the clinical need for more accurate biomarkers to diagnose, monitor, and predict outcomes in JDM is challenging—partly because the role of muscle in initiating and sustaining disease activity remains poorly understood.

Gaps in our understanding of the role that muscle plays in JDM pathology are partly due to the lack of an adequate disease model to test hypotheses and the rarity and heterogeneity of the disease. JDM affects approximately 2–4 children per million in the US (4). Muscle biopsy is not routinely done for diagnosis or prognosis, making the availability of patient samples limited within the US for research purposes (5). Current murine models of myositis also have limitations; there are inherent genomic differences between human and rodent species (6), and no model currently recapitulates myositis development at a young age of onset or exhibits phenotypical and histopathologic hallmarks to mimic JDM (7). To overcome some of these obstacles, we have used a human muscle-derived microphysiological system called myobundles. Myobundles function and respond physiologically to soluble factors, such as type I interferons, mimicking JDM features such as reduced contractility, myositis-specific antigens, and proinflammatory gene response (8, 9). Such an *in vitro* human-based functional model has potential to serve as an effective approach to test hypothesized disease-driving mechanisms, expedite biomarker discovery and examine novel therapies preclinically.

To elucidate the role that muscle plays in JDM pathology, investigate potential biomarkers driven by type I interferonopathy, and further validate the myobundle model of JDM, we conducted a paired basic and translational study of myokine profiles of patient sera and muscle as well as myobundles treated with type I interferons or the interferon stimulant poly(I:C). Myokines are cytokines produced by skeletal muscle. They have diverse effects—both anti-inflammatory and inflammatory—and can perpetuate inflammation as well as muscle weakness through non-inflammatory pathways (10). Myokines have potential as biomarkers of disease activity as well as therapeutic targets in JDM. Through this study, we aimed to define and compare the myokine profiles of myobundles and patients. We measured myokines in the media of healthy pediatric donor-derived myobundles treated with and without type I interferon (either interferon-α or interferon-β), or synthetic viral mimic and interferon stimulant poly(I:C). We then measured and compared myokine profiles of sera from treatment-naïve JDM patients and healthy pediatric patients. We additionally performed bulk mRNA sequencing on patient muscle and myobundles to investigate changes in myokine gene transcription. We hypothesized that IFN and poly(I:C) exposure alters myobundle myokine production, that treatment-naïve JDM patients have altered proinflammatory myokine profiles compared to controls, and that differences in myokine profiles between untreated and treated myobundles are similar to differences between healthy and JDM patients. Here, we show that the myokine profile of JDM patients significantly differs from healthy controls, and that the increase in proinflammatory myokines seen in JDM can be recapitulated in the IFN-stimulated myobundle model via gene and protein expression. Clinical correlation of JDM myokine signature demonstrates proof-of-concept on the utility of myokine profiling to predict strength testing better than traditional muscle enzyme biomarkers and suggests that muscle contributes to aberrant localized and systemic inflammation in JDM patients.

## 2 Material and methods

**Figure 1:**
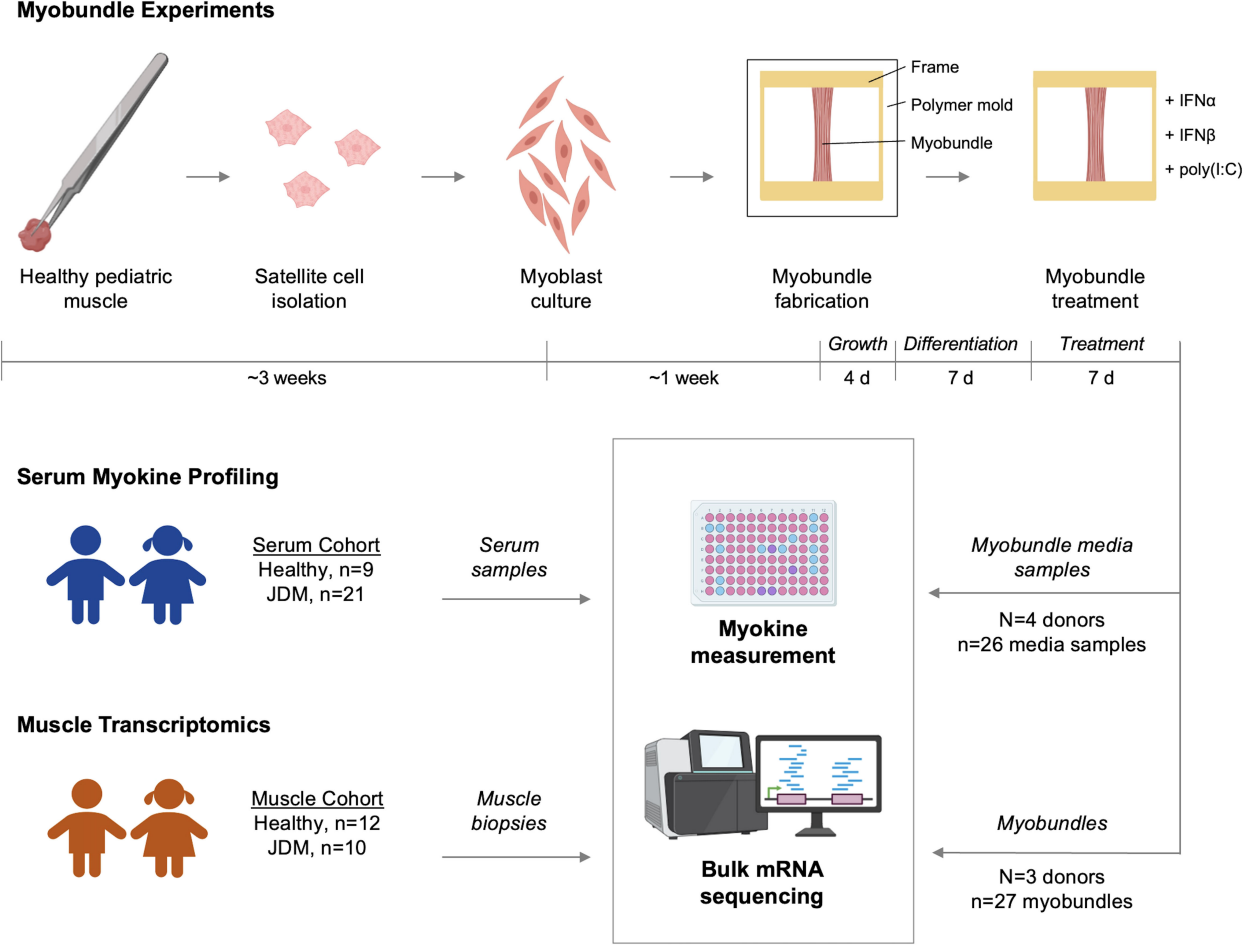
Study design. Myobundles were fabricated from myoblasts isolated from 4 healthy pediatric donors and treated with either IFNα, IFNβ, or IFN stimulant poly(I:C). Media from myobundle culture and flash frozen myobundles were used for myokine measurement and mRNA sequencing, respectively. Healthy and JDM patient serum and muscle biopsies from separate cohorts were collected and used for myokine measurement and mRNA sequencing, respectively.

Overall schema of study methods is depicted in **Figure 1**.

### 2.1 Human primary myoblast isolation and culture

Human skeletal muscle samples were obtained from four healthy pediatric donors as surgical waste from paraspinal muscles during operative treatment for orthopedic non-muscle diagnoses. In our experience, myoblast isolation has been successful, with myobundle contractility and response to IFN remaining consistent between paraspinal muscle waste and quadriceps muscle biopsy sources. Sample collection occurred under Duke University IRB approved protocols. Primary myoblasts were isolated, expanded, and cryopreserved according to previously described methods (11, 12). Briefly, muscle samples were minced, washed in PBS, and enzymatically digested in 0.05% trypsin for 30 min. Muscle was collected by centrifugation, pre-plated for 2 hr, and transferred to a Matrigel (Corning, Glendale, AZ, USA) coated flask for attachment. Cells were expanded in skeletal myoblast growth media containing low glucose DMEM (Thermo Fisher Scientific, Waltham, MA, USA), supplemented with EGF (VWR, Radnor, PA, USA), fetuin (Millipore, Burlington, MA, USA), dexamethasone (Sigma-Aldrich, St. Louis, MO, USA), gentamicin without insulin (Thermo Fisher Scientific, Waltham, MA, USA), amphotericin B (Thermo Fisher Scientific, Waltham, MA, USA) and 10% fetal calf serum (Hyclone, Logan, UT, USA). Myoblasts were cryopreserved in 90% growth medium with 10% DMSO at passage 2 then cultured and used to create passage 4 myobundles.

### 2.2 Human myobundle fabrication and culture

Donor-specific myobundles were fabricated using a protocol previously described (8, 12). Briefly, each myobundle consisted of myoblasts from a single donor (7.5 x 10^5^ cells) mixed with fibrinogen, Matrigel, thrombin, and growth media as described above with addition of 1.5 mg/mL 6-aminocaproic acid (GM). The cell solution was pipetted into custom-made polydimethylsiloxane (PDMS) molds (cast from Teflon masters and pretreated with pluronic) between beams of a porous nylon frame (Cerex Advanced Fabrics, Cantonment, FL, USA). Myobundles polymerized to the frame for 30 min at 37°C before the addition of GM and placement on a rocker at 37°C. GM was exchanged daily for 4 days before myobundle removal from molds and switch to differentiation media containing LG-DMEM, 1% N2 supplement (Thermo Fisher), gentamicin, amphotericin B, and 2 mg/mL 6-aminocaproic acid. Myobundles differentiated over 7 days on a rocker at 37°C with differentiation media exchanged every other day.

### 2.3 Myobundle treatment and culture media collection

After maturation, myobundles were treated with one of the following reagents added to differentiation media exchanged every other day for 7 days: IFNα (Sigma-Aldrich, St. Louis, MO, USA; Interferon-alpha 2A human, 10 µg/mL in 0.1% BSA in PBS), IFNβ (PeproTech, Cranbury, NJ, USA; 10 µg/mL in 0.1% BSA in PBS), or synthetic dsRNA poly(I:C) (Invivogen, San Diego, CA, USA; 10 µg/mL in water). Myobundles were treated with IFNα or IFNβ diluted to 0 (control), 5, 10, or 20 ng/mL or poly(I:C) at 10 µg/mL every other day for 7 days. There is a paucity of literature on the concentration of IFN found in JDM muscle due to technical and biological limitations, including the cytokines’ nonspecific immunoreactivity, transient expression, and presence at low concentrations (13, 14). Thus, the concentration of IFN used was based on dose-dependent responses observed in myobundle contractility in prior work (8) and informed by IFNγ concentrations used in prior myobundle study, in which up to 20 ng/mL was used (15). On the seventh day of treatment (Day 18 from initial myobundle creation), 1 mL aliquots of myobundle culture media were collected from all treatment conditions and frozen at -80°C until re-aliquoting for sample preparation.

### 2.4 Patient sera, muscle biopsy, and clinical data collection

Blood samples were collected from healthy patients younger than 18 years old and patients with newly diagnosed, treatment naïve JDM within the Duke Children’s Myositis Center under IRB approved protocols. Muscle biopsies were collected via open surgical biopsies from the vastus lateralis muscle of JDM and healthy pediatric patients within the multidisciplinary juvenile myositis programs at Duke University and the University of Michigan Ann Arbor. All muscle biopsies were collected as part of standard of care for a clinical diagnostic purpose; excess muscle tissue was collected for this study under IRB approved protocols. Healthy control muscle samples were collected from patients undergoing open surgical biopsy for reasons other than presumed inflammatory or neuromuscular myopathy and were determined to not have JDM by a histopathologist. The cohort of patients whose blood was sampled was distinct from the cohort who received muscle biopsy in exception of 3 JDM patients from Duke. Inclusion criteria for JDM patients was: 1. Under the age of 18 years old; 2. Diagnosis of JDM by a pediatric rheumatologist based on typical disease manifestations (16–18); and 3. Not yet treated with immunosuppressive therapy. Demographic and clinical data including laboratory markers, such as myositis-specific antibody status, and functional measures of strength were collected from the timepoint of sera and/or muscle collection.

### 2.5 Myokine measurement

Quantification of myokines was performed within the Biomarkers Core Facility at the Duke Molecular Physiology Institute. Myobundle supernatant samples were run for singlicate analyses; Patient serum samples were run for duplicate analyses. Frozen samples were first thawed on ice. Total volumes of 280 µL per supernatant sample and 140 to 155 µL per serum sample were used for all assays. MSD Custom Human UPLEX Assay (Cat#K151AEM, Cat#K15067M Meso Scale Diagnostics, Rockville, MD, USA) was used to measure IL-6, IL-8, IL-17A, TNFα, CXCL10, IFN-α2a, IL-15, IL-18, CXCL9, and RANTES. IFNβ, CXCL2, and decorin were measured with MSD SPLEX Human IFN-β (Cat#K151ADRS, MSD), human MIP2 ELISA (Cat#ab184862, Abcam, Cambridge, UK), and human Decorin ELISA (Cat#ab99998, Abcam), respectively. Due to the high sensitivity of the SPLEX IFNβ assay, supernatant samples from myobundles treated with IFNβ were run at a 10-fold dilution, while all other myobundle supernatant samples were run at a 2-fold dilution for this assay.

### 2.6 RNA sequencing

RNA extraction from flash frozen myobundles and approximately 5 mg of muscle tissue per patient biopsy was performed using a RNeasy Mini Kit (Qiagen catalog#74134, Hilden, DE). Samples were stored at -80°C until assayed. RNA libraries were built by the Duke Sequencing and Genomics Technologies Core Facility using stranded mRNA kit (Roche: 07962207001), and libraries were sequenced as 50 pair-ended base pairs with a NovaSeq 6000 S-Prime. RNA-seq data was processed as previously described (9). RNA-seq data was processed using the fastp toolkit (19) to trim low-quality bases and sequencing adapters from the 3’ end of reads, then mapped to GRCh38 (downloaded from Ensembl, version 106) (20) using the STAR RNA-seq alignment tool (21), and reads aligning to a single genomic location were summarized across genes. We combined technical replicates using the DESeq2 (22) Bioconductor (23) package implemented for the R programming environment. Following DESeq2 recommendations, we removed genes from our analyses if a given gene has less than 10 total mapped reads in at least three samples. The remaining gene count data were normalized using DESeq2. The log_2_-transformed normalized count data discussed in this publication is accessible in **Supplementary Table 1**.

### 2.7 Statistical analysis

Results were analyzed and visualized with GraphPad Prism (version 10.4.1), JMP Pro 17 and R. For analysis of myobundle media and serum myokine measurements, Prism was used for one-way ANOVA with Tukey *post-hoc* multiple comparisons and Mann-Whitney U test as appropriate. Principal Component Analysis (PCA) with loading matrix calculation of sera and media myokine data was performed using JMP Pro 17. Z-scores of media and sera data were calculated as [mean(treatment group)-mean(myokine)]/SD(myokine), and heatmaps with hierarchical clustering were created using R. Multivariate linear regression was conducted with Prism to assess the relationships between biomarker data and CMAS as well as between myobundle and patient myokine measurements. Log_2_-transformation was implemented prior to one-way ANOVA analysis of normalized gene mRNA expression of myokines in muscle biopsies and myobundles. Adjusted p values for the false discovery rate were determined using an R program (p.adjust) that applied the method of Benjamini and Hochberg (24).

## 3 Results

### 3.1 Interferon and poly(I:C) lead to distinct pro-inflammatory myokine response

**Figure 2:**
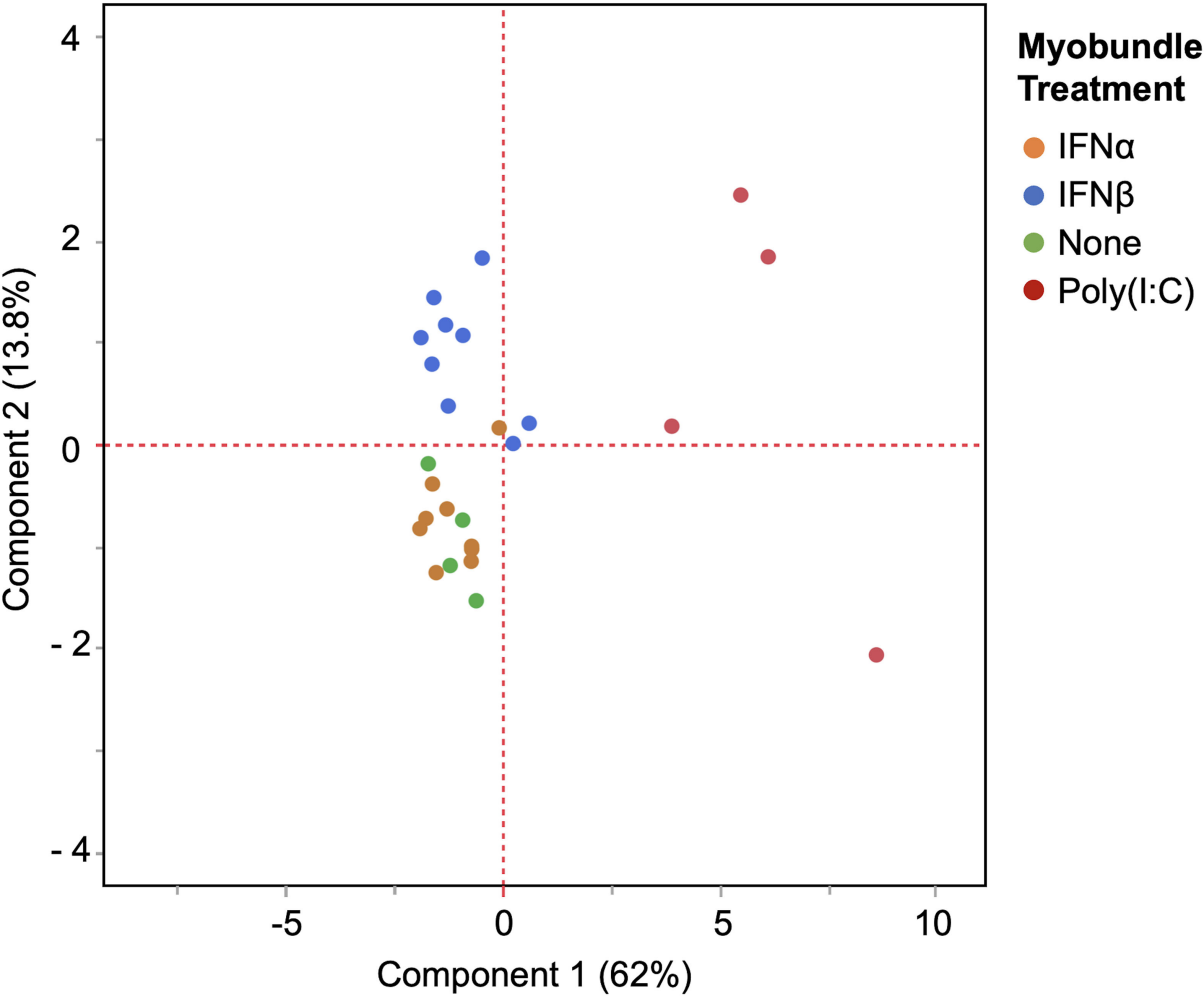
Poly(I:C) leads to greatest change in pro-inflammatory myokines. Principal Component Analysis of myobundle myokine profiles shows greatest shift along Principal Component 1 from poly(I:C) treatment, followed by IFNβ treatment. IFNα-treated myobundles cluster near untreated control myobundles.

Principal Component Analysis conducted on myokine profiles across myobundles demonstrates that poly(I:C) led to the greatest overall degree of change in myokine response compared to untreated controls (**Figure 2**). Principal component 1 (PC1) accounted for 62% of variance and separates poly(I:C) and IFNβ-treated myobundles into distinct clusters. IFNα-treated myobundles cluster amidst untreated controls.

**Figure 3:**
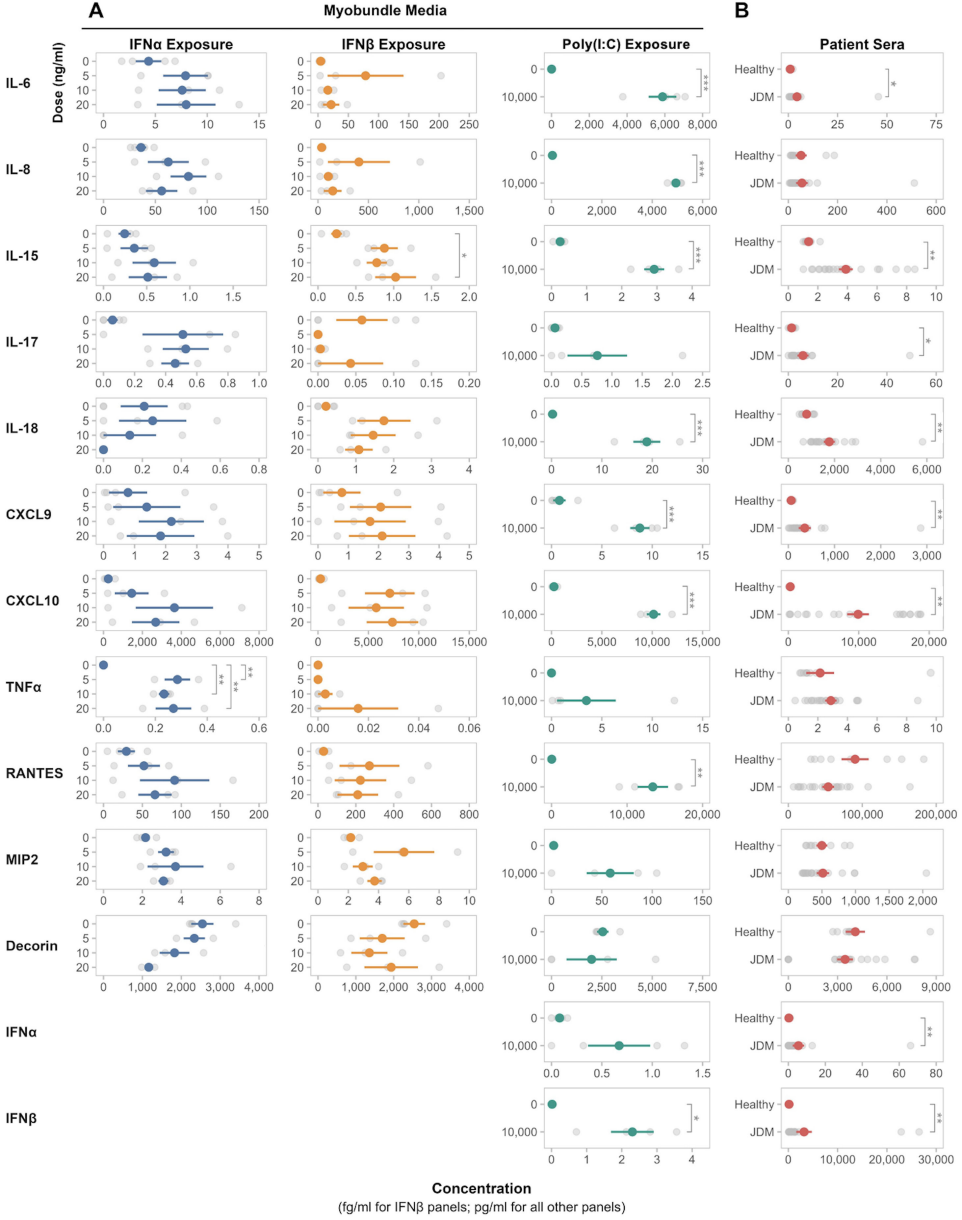
Myokine profiles in myobundle media and JDM patient sera. Myokine profiles from media of myobundles **(A)** after exposure to various concentrations of IFNα, IFNβ, and poly(I:C) and in treatment-naïve JDM patient sera **(B)**. Myobundles derived from 3–4 healthy pediatric donors; n=2–8 myobundles per donor. For patient sera samples, healthy controls n=9, JDM n=21. Mean ± SEM is shown. *P_adj_ ≤ 0.05; **P_adj_ ≤ 0.01; ***P_adj_ ≤ 0.001.

From analysis of individual myokine response of myobundles to IFN stimulation, IFNα treatment of healthy myobundles across 3 pediatric donors led to a significant increase in TNFα production after 5, 10, and 20 ng/mL exposure (p_adj_=0.004). IFNα at the 20 ng/mL concentration also led to a dose-dependent decrease in decorin, although this change did not reach statistical significance (p_adj_=0.17). All other myokines measured after IFNα exposure revealed no significant change compared to untreated myobundles. Furthermore, IFNα itself as a myokine was undetectable in the media of myobundles treated with IFNβ and poly(I:C) (**Figure 3A**).

In contrast to IFNα-induced changes in TNFα production by myobundles, IFNβ treatment did not lead to significant change in TNFα, yet did induce significant increase in IL-15 (p_adj_ =0.03) and numerical, yet not statistically significant, increases in CXCL9 and CXCL10. (**Figure 3A**).

Poly(I:C) treatment of healthy myobundles led to the most significant pro-inflammatory response in myokine production. IL-6, IL-8, IL-15, IL-18, CXCL9, CXCL10, RANTES, and IFNβ were all significantly increased in the media of poly(I:C)-treated myobundles compared to untreated myobundles across 4 healthy pediatric donors (p_adj_<0.001-0.01) (**Figure 3A**).

### 3.2 JDM patients have heterogenous and altered pro-inflammatory myokine profiles

Serum samples from 21 treatment-naïve, newly diagnosed JDM patients were compared to those of 9 healthy pediatric controls. **Table 1** lists the demographic and clinical features of this JDM patient serum cohort with **Supplementary Text S1** describing specific details of myositis-specific and myositis-associated antibody status.

**Table 1:** Demographic and clinical features of treatment-naïve JDM patient cohort from which serum samples were collected.

Cohort for Patient Sera	JDM (n=21)	Missing data (n,%)
Age, yr ( ± SEM)	8.9 (1.1)	
Female, n (%)	17 (81.0%)	
Race & ethnicity, n (%)		
Non-Hispanic White	11 (52.4%)	
Hispanic	5 (23.8%)	
Black	4 (19.0%)	
Unknown	1 (4.8%)	
CMAS ( ± SEM)	29.3 (3.2)	3 (14.3%)
CDASI-A ( ± SEM)	8.2 (2.1)	12 (57.1%)
MSA positive, n (%)	13 (61.9%)	
NXP-2	4 (19.0%)	
Mi-2	1 (4.8%)	
Tif-1γ	3 (14.3%)	
MDA5	1 (4.8%)	
Multiple	3 (14.3%)	Mi-2/Tif-1γ (2)
		NXP2/Tif-1γ (1)
Other	1 (4.8%)	PL-12
None	8 (38.1%)	
MAA positive, n (%)	5 (23.8%)	
Anti-SSA/Ro	2 (9.5%)	
RNP	2 (9.5%)	
Other	2 (9.5%)	ACA, PM/SCL
Physician’s Global ( ± SEM)	5.4 (0.6)	12 (57.1%)
PGA muscle	3.7 (0.7)	12 (57.1%)
PGA skin	3.6 (0.7)	12 (57.1%)
PGA extramuscular (no skin)	1.9 (0.7)	12 (57.1%)
Patient/Parent Global	6.7 (0.9)	18 (85.7%)
Mean serum muscle enzymes (+SEM)		
AST (U/L)	154.7 (36.4)	1 (4.8%)
LDH (U/L)	442.6 (76.2)	
Aldolase (U/dL)	34.1 (8.5)	
CK (U/L)	2167.5 (1070.5)	1 (4.8%)

Compared to healthy controls, JDM patients had significantly elevated levels of IL-6, IL-15, IL-17, IL-18, CXCL9, CXCL10, IFNα and IFNβ (p_adj_ =0.001-0.03). There were no significant differences in the measurement of IL-8, TNFα, RANTES, MIP2 (CXCL2), or decorin between JDM and healthy patients (**Figure 3B**).

**Figure 4:**
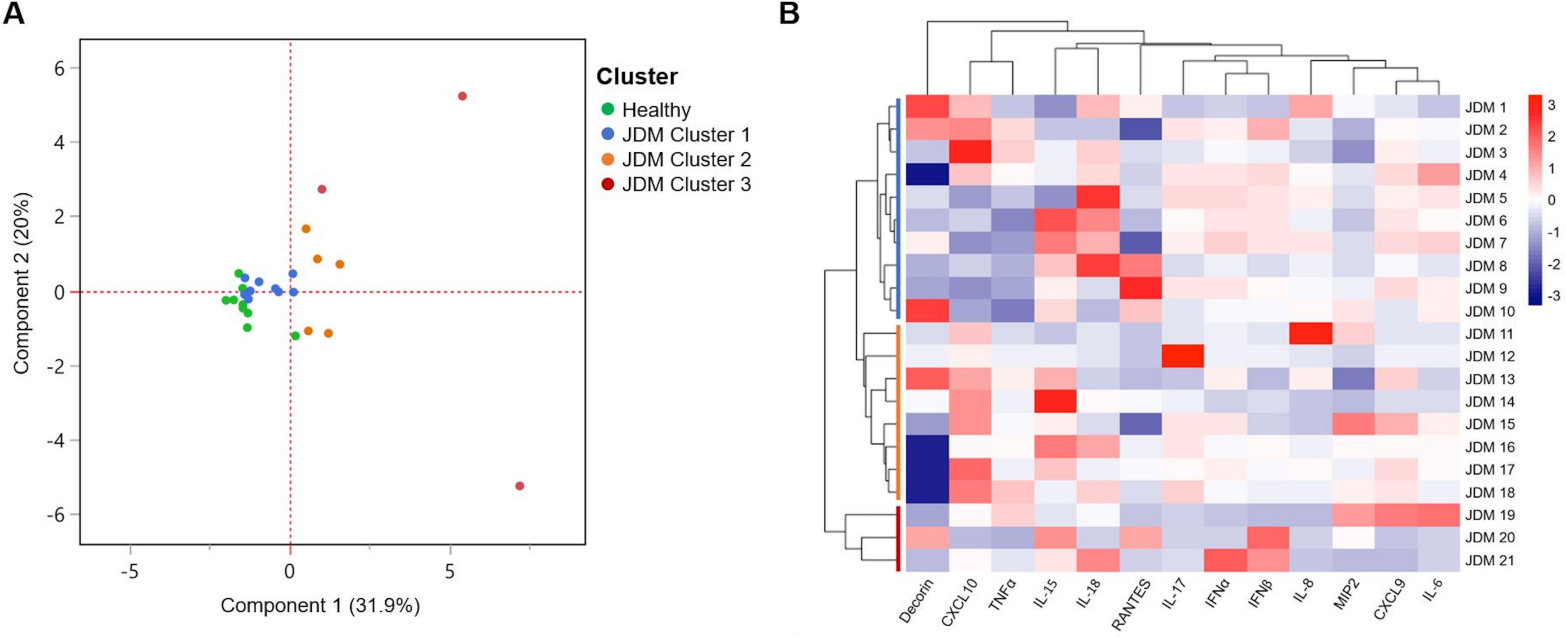
JDM patient serum myokine profiles. **(A)** Principal Component Analysis of myokine profiles in patients separates healthy controls from JDM patients, which separated into three distinct clusters. **(B)** Heatmap comparing Z-score of myokines measured across JDM patients reveals notable heterogeneity, including outliers within cluster 3.

To further characterize myokine profiles of patients, Principal Component Analysis was performed across all patients (**Figure 4A**). Healthy patients separated from JDM patients along PC1, which accounted for 31.9% of variance. PCA and hierarchical cluster analysis revealed three distinct clusters of JDM patients based on their myokine profiles and calculated z-scores (**Figure 4B**). There was notable heterogeneity across JDM patient profiles. One cluster, Cluster 3, included three outlier patients (patients 19, 20, and 21) that had significantly different profiles than that of the other two (**Figure 4**). Despite significant heterogeneity, Cluster 1 was noted to have more consistently elevated IL-18; Cluster 2 had higher levels of CXCL10. Comparison of clinical disease severity between clusters was limited due to the small number of patients within each cluster and missing clinical data, such as Physician Global Assessment scores. However, there was no statistically significant difference between clusters in patient age, CMAS, or conventional muscle enzymes (AST, LDH, aldolase, or CK) based on one-way ANOVA analysis (**Supplementary Figure 1**).

### 3.3 Multivariate regression reveals select myokines as predictors of clinical strength

**Figure 5:**
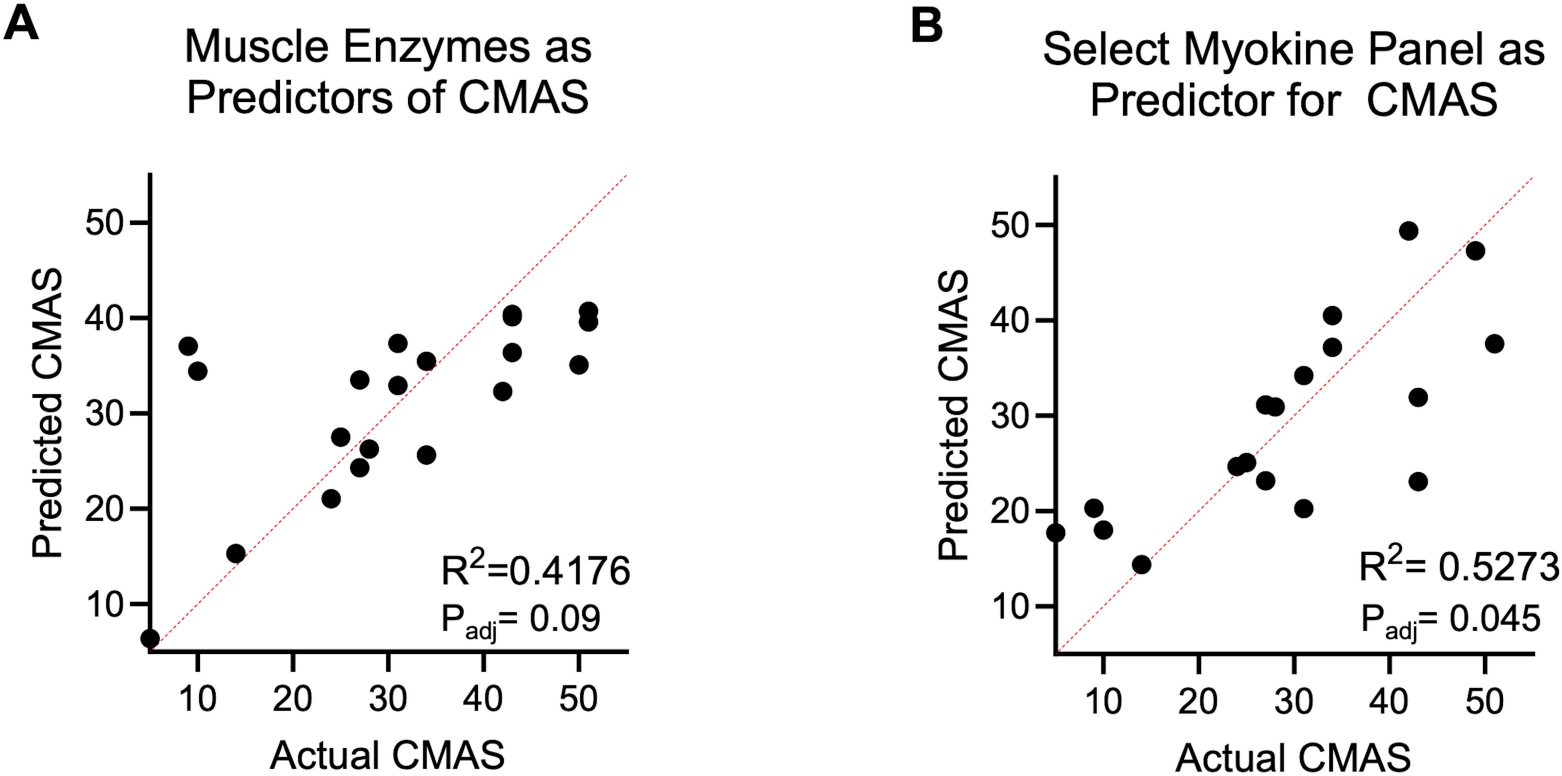
Multivariate linear regression of biomarkers as predictors of CMAS. **(A)** Regression analysis of muscle enzymes LDH, CK, aldolase, and AST in relation to CMAS. **(B)** Regression analysis of a select myokine panel (including IL-6, IL-15, IL-18, CXCL9, CXCL10) measured in patient sera based on PCA of patient sera profiles as predictors of CMAS.

**Table 1** lists average values of conventional markers of muscle disease activity in the JDM patient serum cohort. Mean values of AST, LDH, aldolase, and CK were elevated above normal reference ranges, consistent with active muscle disease. Multivariate linear regression was conducted to examine the relationship between these four conventional muscle enzyme labs and CMAS score. Although the model explained approximately 42% of variance in CMAS (R^2^ = 0.42), it did not reach statistical significance (p_adj_=0.09), and none of the lab markers were individually significant (**Figure 5A**).

The PCA of healthy and JDM patient sera data shown in **Figure 4A** revealed variables contributing strongly to PC1 included CXCL9, CXCL10, IL-6, IL-15, and IL-18. Thus, multivariate linear regression analysis was conducted to assess the relationship between a panel including these 5 myokines (CXCL9, CXCL10, IL-6, IL-15, and IL-18) and CMAS scores. The model explained a higher percent of variance in CMAS than the combination of AST, LDH, aldolase and CK (R^2^ = 0.53) and met statistical significance (p_adj_=0.05) (**Figure 5B**). Through the same multivariate regression analysis, IL-6, CXCL9, and CXCL10 were found to all be statistically significant independent predictors of CMAS (p_adj_=0.03, 0.03, 0.02, respectively). IL-15 and IL-18 did not show significant independent effect on CMAS score.

### 3.4 Transcriptional myokine profile of myobundles mimics that of JDM muscle

**Figure 6:**
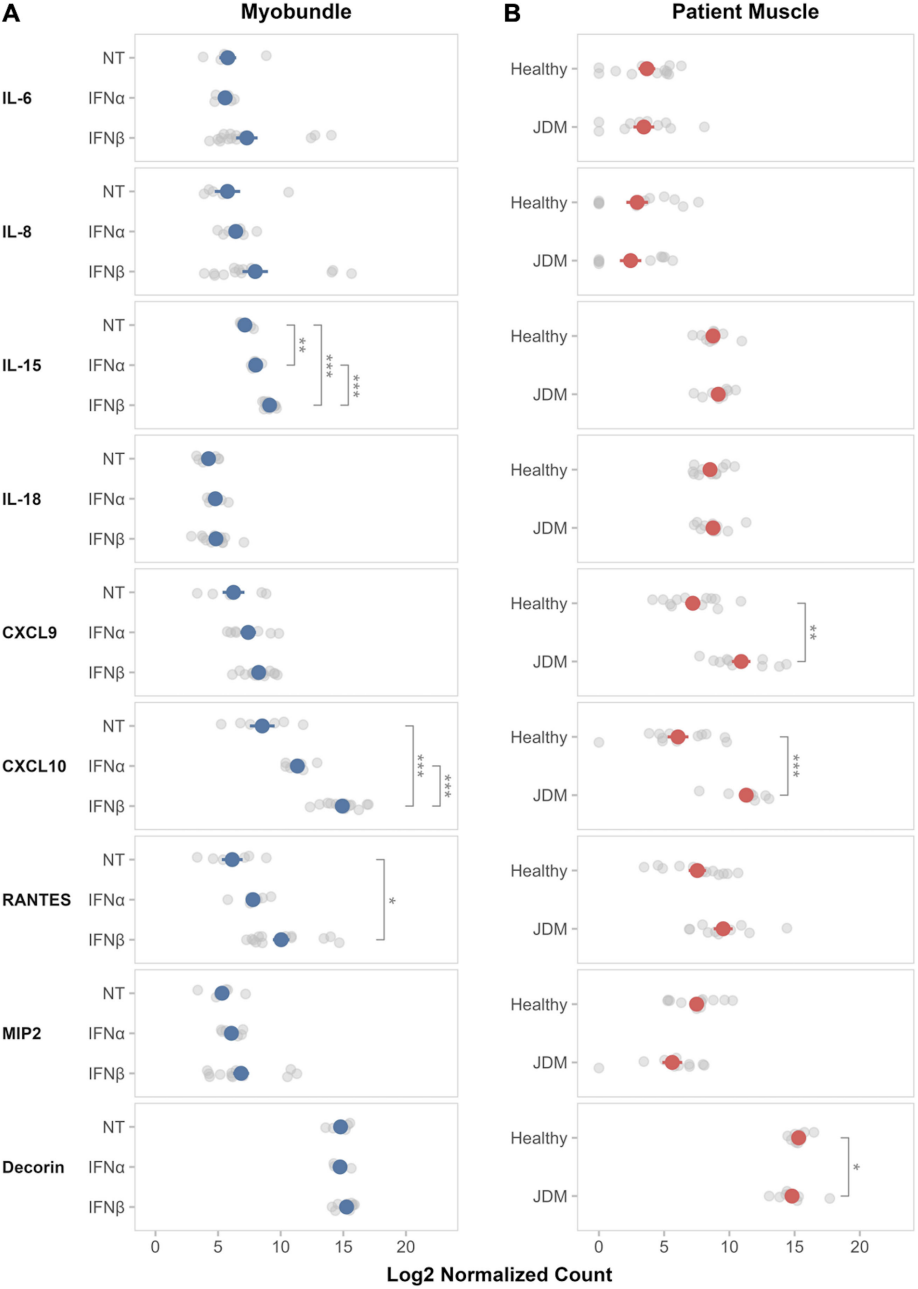
Comparison of myokine transcriptional response in IFN-treated myobundles and JDM muscle biopsies. **(A)** RNA sequencing analysis of protein-encoding genes of myokines in healthy pediatric donor-derived myobundles (N = 3 donors, n=8–11 myobundles per donor) compared to **(B)** RNA sequencing analysis of similar genes in healthy and JDM patient muscle biopsies (Healthy, n=12; JDM, n=10). *P_adj_ ≤ 0.05; **P_adj_ ≤ 0.01; ***P_adj_ ≤ 0.001.

Untreated and IFNα/IFNβ-treated myobundles underwent mRNA sequencing to orthogonally test transcriptional response to IFN for the 13 myokines measured in the supernatant. Of the 13 myokines, 9 had a singular responsible protein-encoding gene that had greater than 10 copies across more than three samples. Protein-encoding genes for IFNα, IFNβ, IL-17A, and TNFα had less than 10 copies across more than three samples so were not included in analysis. The genes were analyzed across treated myobundles derived from 3 healthy pediatric donors. All doses of IFNα and IFNβ were pooled into an IFNα-treated and IFNβ-treated group, respectively. Log_2_-transformed normalized counts were used for analysis. IFNα exposure was associated with numerical increase in CXCL10 (p_adj_=0.08) and significant increase in IL-15 (p_adj_=0.005) expression compared to untreated myobundles, which was not seen in myobundle media as a protein response. This may be due to IFNα-induced change in post-transcriptional regulation (25, 26), post-translational regulation (27) or limitations in the sensitivity of protein detection by UPLEX assay. IFNβ exposure was associated with greater increase in CXCL10 and IL-15 gene expression (p_adj_ <0.001), along with significant increase RANTES (p_adj_ =0.03). CXCL9 numerically increased in both IFNα- and IFNβ-treated myobundles, yet did not reach statistical significance. While IL-15 measured in media correlates with this increase in gene expression, CXCL9, CXCL10 and RANTES measurement in media numerically increased after IFNβ treatment but did not reach statistical significance. (**Figure 6A**).

Bulk RNA-sequencing data from the muscle biopsies of 10 treatment-naïve JDM patients and 12 healthy pediatric control patients was also analyzed. Demographic and clinical data of the JDM cohort are listed in **Table 2**. Average age of the muscle biopsy JDM cohort was 9 (± 1.2) years with majority (60%) female and non-Hispanic white (40%). Average CMAS was 35 (± 6.2), indicating moderate disease severity, although it is noted that 40% of the cohort did not have a recorded CMAS around the time of biopsy. Average PGA score was 6.4 (± 0.8) out of a maximum score of 10. Forty percent of patients had MSA positivity with 3 of the 4 patients being anti-MJ/NXP-2 positive.

**Table 2:** Clinical and demographic data of the treatment-naïve JDM patient cohort from which muscle biopsies were obtained.

Cohort for muscle biopsy	JDM (n=10)	Missing data (n,%)
Age, yr ( ± SEM)	9 (1.2)	
Female, n (%)	6 (60%)	
Race & ethnicity, n (%)		
Non-Hispanic White	4 (40%)	
Hispanic	2 (20%)	
Black	2 (20%)	
Other	1 (10%)	Black/White
Unknown	1 (10%)	
CMAS ( ± SEM)	35 (6.2)	4 (40%)
CDASI-A ( ± SEM)	5 (2.3)	4 (40%)
MSA positive, n (%)	4 (40%)	2 (20%)
NXP-2	3 (30%)	
Mi-2	1 (10%)	
None	4 (40%)	
MAA positive, n (%)	2 (20%)	3 (30%)
Anti-SSA/Ro	2 (20%)	
Physician’s Global ( ± SEM)	6.4 (0.8)	3 (30%)
PGA muscle	5.7 (0.3)	7 (70%)
PGA skin	3.7 (2.0)	7 (70%)
PGA extramuscular (no skin)	2.7 (1.5)	7 (70%)
Patient/Parent Global	6.3 (1.2)	4 (40%)
Mean serum muscle enzymes (+SEM)		
AST (U/L)	94.7 (34.0)	1 (10%)
LDH (U/L)	416.3 (53.9)	1 (10%)
Aldolase (U/dL)	24.6 (5.9)	1 (10%)
CK (U/L)	691.4 (338.3)	1 (10%)

In patient muscle biopsies, the same 9 myokine genes were analyzed similarly to myobundle samples. Compared to healthy muscle biopsies, JDM muscle showed increased CXCL10 and CXCL9 gene expression (p_adj_<0.001-0.003), mirroring the higher levels of CXCL10 and CXCL9 in peripheral serum. This may indicate that muscle is a contributing source to pro-inflammatory signaling systemically. Interestingly, decorin gene expression was significantly decreased in JDM muscle (p_adj_=0.05), yet there was no difference between healthy patient and JDM sera. In addition, elevated levels of IL-15 and IL-18 observed in JDM patient sera were not seen in IL-15 and IL-18 gene expression of muscle biopsies. This discordance may be explained by the larger contribution of IL-15 and IL-18 by circulating immune cells (28) or local post-transcriptional regulatory mechanisms that promote mRNA degradation (29). Of note, the patient cohorts used in the peripheral sera collection and muscle biopsies only had 3 overlapping JDM patients, precluding direct comparisons (**Figure 6B****).**

Comparing the myobundle model to patient data, both CXCL9 and CXCL10 gene expression were increased in both IFN-treated myobundles and JDM muscle biopsies, compared to their respective untreated and healthy controls (**Figure 6**).

### 3.5 IFN-stimulated myobundle signature recapitulates key aspects of JDM myokine profile

When comparing myokine profiles of the myobundle model with patients, 6 of 13 measured myokines (IL-6, IL-15, IL-18, CXCL9, CXCL10, and IFNβ) were significantly increased in both JDM patient sera and treated myobundle media compared to controls. Both IFNβ and poly(I:C) induced increased IL-15 production, and poly(I:C) alone induced increased levels of IL-6, IL-18, CXCL9, CXCL10, and IFNβ. CXCL9 and CXCL10 showed consistent increase in treated myobundle media and JDM patient sera, as well as in gene expression in treated myobundles and JDM muscle, compared to controls.

**Figure 7:**
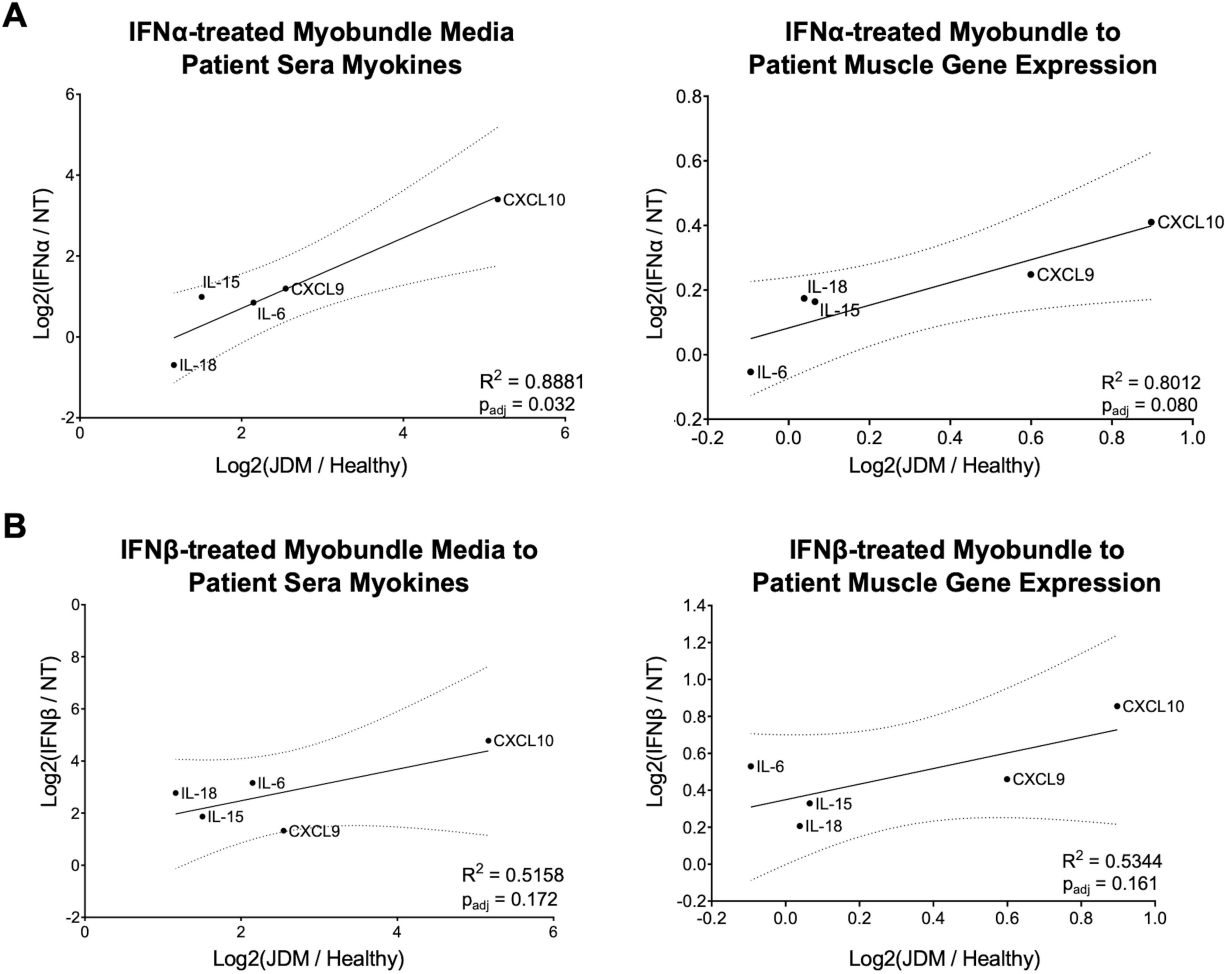
Correlation of IFN-treated myobundle model with JDM patient sera and muscle gene expression. Simple linear regression was used to compare log2-fold change of select myokines (CXCL9, CXCL10, IL-6, IL-15, and IL-18) in treated myobundles over untreated controls and JDM patients over healthy controls. Myokine production and gene expression of IFNα-treated myobundles had a positive association with JDM patient log2-fold changes **(A)**. IFNβ-treated myobundles also had positive association with JDM patient sera and muscle gene expression **(B)** yet did not reach statistical significance.

To further compare the myobundle model to patients, we first calculated the log2-fold change of each myokine’s mean gene expression and protein production of treated or diseased state over controls. Simple linear regression was then used to examine the relationship between patient log2FC(JDM/healthy) to myobundle log2FC(treated/untreated) protein and gene expression. Inclusion of all 13 myokines in simple linear regression showed a significant positive association between IFNα-treated myobundles and JDM patients, in media and sera myokine values (R^2^ = 0.48, p_adj_=0.02), yet no significant relationship between IFNβ-treated or poly(I:C)-treated myobundles with JDM patients (**Supplementary Figure 2**). When we included only the five myokines that strongly contributed to PC1 of the PCA of patient sera (CXCL9, CXCL10, IL-6, IL-15, and IL-18), the positive association between myobundles and JDM patients was strengthened. For IFNα-treated myobundle media correlation to patient sera log2FC, there was a significant positive association (Y = 0.8723*X – 1.037, p_adj_=0.03, R^2^ = 0.89, 95% confidence interval for the slope ranged from 0.3033 to 1.441). This positive relationship was mirrored in myokine gene transcription of IFNα-treated myobundles and JDM patient muscle, although it did not reach statistical significance (Y = 0.3519*X + 0.08263, p_adj_=0.08, R^2^ = 0.80, 95% confidence interval for the slope ranged from 0.02987 to 0.6739) (**Figure 7A**). Simple linear regression of the same 5 myokines (CXCL9, CXCL10, IL-6, IL-15, and IL-18) in IFNβ-treated myobundles to JDM patients revealed a positive association, yet not statistically significant in both media and sera correlation (Y = 0.6048*X + 1.265, p_adj_=0.17, R^2^ = 0.52, 95% confidence interval for slope ranged from -0.4719 to 1.682) and gene transcription in myobundles and muscle (Y = 0.4220*X + 0.3491, p_adj_=0.16, R^2^ = 0.53, 95% confidence interval for slope ranged from -0.3017 to 1.146) (**Figure 7B**).

### 3.6 Differences between myobundle and patient profiles highlight IFN-induced muscle inflammation

In comparing the myokine profiles of myobundles with that of patients, 5 of the 13 myokines measured did not correlate in differences seen between treated or diseased state to controls. First, levels of IL-8 and RANTES were significantly elevated in the media of myobundles treated with poly(I:C) yet did not differ between JDM and healthy patient sera. Such discrepancies suggest that muscle cells produce IL-8 and RANTES in response to strong IFN stimulation, but these changes at the muscle tissue level are not reflected in systemic circulation. Second, TNFα levels significantly increased after IFNα exposure of myobundles, but numerical increase between JDM and healthy patient sera did not reach statistical significance. This difference may be due to inadequate statistical power of our small patient sample size or may reflect local change in muscle microenvironment that is not fully detected on a systemic circulatory level.

In contrast, IL-17 and IFNα were the only 2 myokines measured that showed significant elevation in JDM patient sera compared to controls, yet not in the media of treated myobundles. Although poly(I:C)-treated myobundles were associated with numerically increased levels of both IL-17 and IFNα compared to controls, the changes did not reach statistical significance. This demonstrates that while IL-17 and IFNα can be produced by muscle cells in response to IFN stimulation, other cell types such as peripherally circulating immune cells generate IL-17 and IFNα to a greater degree. In fact, of the myokines measured in this study, IL-6, IL-8, IL-15, RANTES, and TNFα have been shown to be produced by muscle cells in both basal and inflammatory conditions (30–32). The remaining myokines measured (IL-17A, IL-18, CXCL9, CXCL10, IFN-α2a, IFNβ, CXCL2, and decorin) can be produced by muscle yet are primarily derived from other cell types *in vivo*. These cell types include Th17 cells (source of IL-17A), monocytes (IL-18, CXCL2, CXCL9, CXCL10), macrophages (IL-18, CXCL2), dendritic cells (IL-18, IFN-α2a, IFNβ), endothelial cells (CXCL2, CXCL9, CXCL10, decorin), fibroblasts (decorin, IFNβ), and smooth muscle cells (decorin) (33–35).

## 4 Discussion

We defined a pro-inflammatory, heterogeneous myokine signature in both blood and muscle of treatment-naïve JDM patients and showed that a bioengineered human muscle model can recapitulate this signature through type I interferon pathway activation. In this model, myobundles displayed an inflammatory response to IFNα, IFNβ, and poly(I:C). In analysis of the myokines that most strongly contributed to variance in patient serum (CXCL9, CXCL10, IL-6, IL-15, and IL-18), IFNα treatment of myobundles produced the strongest correlation with JDM patient serum and muscle gene expression profiles; poly(I:C) triggered the highest levels of pro-inflammatory myokine release. These findings indicate that IFN pathway activation in muscle can drive both local and systemic inflammation, potentially fueling disease activity and linking directly to clinical muscle weakness. Further, this work demonstrates the utility of the myobundle platform as a powerful tool to shed light on JDM pathogenesis and to investigate biomarkers that may surpass conventional laboratory tests in predicting disease activity.

Of the myokines measured in this study, CXCL9 (MIG) and CXCL10 (IP-10) showed the most consistent and significant increase compared to controls; they were upregulated across IFN-stimulated myobundle media, JDM patient sera, and RNA gene transcription in both myobundles and patient muscle. Serum CXCL9 and CXCL10 levels were also statistically significant, independent predictors of JDM patient CMAS scores. Both are interferon-γ-stimulated chemokines that play a role in interferon-driven inflammation, including Th1 immune response and recruitment of macrophages, NK cells, and plasmacytoid dendritic cells (35). Of the myokines measured in this study, CXCL9 and CXCL10 have had the most robust evidence thus far as diagnostic markers, monitoring disease activity and response to treatment (36–38). Both are upregulated in lesional skin of JDM patients (39) and their receptor, CXCR3, is overexpressed in perifascicular infiltrates of dermatomyositis muscle, implicating a role in recruiting IFN-producing plasmacytoid dendritic cells to tissue (40). Furthermore, elevated or rising CXCL10 serum level may precede disease flare in the absence of CK elevation in JDM patients (41). Here, we not only corroborate prior findings of clinically relevant, elevated levels of CXCL9 and CXCL10 in JDM patients, but we also demonstrate that their gene expression and production can be induced *in vitro* using human bioengineered myobundles. Using this model, CXCL9 and CXCL10 may serve as useful surrogates for assessing anti-IFN treatment efficacy in preclinical drug testing.

Similar to CXCL9 and CXCL10, the three cytokines IL-6, IL-15, and IL-18 were significantly elevated in active JDM patient sera, strongly contributed to variance in sera Principal Component Analysis, and were found to be increased in the IFN-stimulated myobundle model as well. This suggests that muscle may be contributing to systemic inflammation in active JDM. IL-6, IL-15, and IL-18 have been noted to be elevated systemically in patients with idiopathic inflammatory myopathies (IIM) (42–44). Both IL-6 and IL-15 have a beneficial, anti-inflammatory effect when produced acutely by skeletal muscle after exercise, however are thought to be detrimental to muscle strength and function when chronically elevated (10, 45). For instance, while a low IL-15 level enhances muscle glucose uptake and mitochondrial oxidative function, chronic IL-15 elevation decreases electron transport chain formation (46). IL-6, which has correlated with disease activity and type I IFN gene signature in dermatomyositis (47, 48), was shown here to be a statistically significant independent predictor of CMAS in our JDM sera cohort. IL-6 is essential for Th17 differentiation (49) and also acts in an endocrine fashion in regulating metabolic function in many organs, including the pancreas, gut, fat and liver. Finally, IL-18 has also been seen to be overexpressed in both serum and muscle of IIM patients with a decrease in levels corresponding to therapeutic response (50, 51). In our study, given consistent upregulation of IL-6, IL-15, and IL-18 in JDM patient serum and media of IFN-stimulated myobundles, future study of these specific myokines as potential biomarkers and mechanistic drivers of JDM is warranted.

A key advantage of the myobundle model is its ability to isolate muscle-intrinsic inflammatory responses from systemic contributors in JDM. Among myokines differing between JDM patients and IFN-stimulated myobundles, IL-8 and RANTES (CCL5) were markedly increased after poly(I:C) stimulation, with RANTES gene expression also elevated after IFNβ treatment and trending higher in JDM muscle. Neither was significantly altered in patient sera, suggesting localized muscle production from IFN pathway activation. Both IL-8 and RANTES are potent recruiters of immune cells; IL-8 promotes neutrophilic inflammation (35), while RANTES is essential for the migration of effector and memory T cells and monocytes to inflamed tissue via CCR5 (52). CCR5 has been linked to MHC I–overexpressing myotubes under ER stress and to inflammatory infiltrates in IIM (53, 54). While RANTES has not been detected by western blot or immunocytochemistry in muscle specimens of IIM in a prior study (55), our data show IFN-stimulated RANTES production detectable by UPLEX, implicating a role in local recruitment of inflammatory cells.

In contrast, IL-17 was elevated in JDM sera but not in JDM muscle or the myobundle model, indicating an extramuscular source. Of note, myobundles do not incorporate immune cells, so this could explain the lack of IL-17 in myobundle media. IL-17 is produced mainly by Th17 cells and neutrophils (56) and correlates with dermatomyositis activity (57, 58), can induce IL-6 release (59), and impair differentiation in myoblasts (60). Its elevation in our cohort aligns with prior reports of abundant Th17+ T cells in JDM muscle (61) and stronger Th17-related gene expression versus adult DM (61). Collectively, these findings highlight the myobundle model’s utility in distinguishing muscle-derived from systemic inflammatory mediators in JDM pathology.

Myobundles exhibited varying degrees of inflammation in response to IFNα, IFNβ, and poly(I:C) treatment. Poly(I:C) induced the most robust inflammatory response, likely because it activates intracellular pattern-recognition receptors, leading to the production of endogenous IFN and subsequent induction of IFN-stimulated gene expression. In addition to this IFN-dependent pathway, poly(I:C) also triggers IFN-independent signaling, which results in a broader inflammatory response than exogenous IFN stimulation alone (62, 63). These signaling cascades may explain why the myokine response of poly(I:C)-treated myobundles showed the weakest correlation with that observed in JDM patient sera (**Supplementary Figure 2**). In contrast, both IFNα- and IFNβ-treated myobundles showed some concordance with JDM serum and muscle myokine profiles; IFNα treatment most closely mirrored the patient cohort. Previously, we demonstrated that IFNβ—but not IFNα—induces a detrimental reduction in contractile force in healthy myobundles (8). Here, we find that while both type I interferons elicit a pro-inflammatory myokine response, IFNα uniquely drives a profile that more closely recapitulates JDM-associated inflammation. Thus, the myobundle model could be a powerful tool to dissect muscle-intrinsic mechanisms contributing to JDM pathogenesis and evaluate targeted anti-interferon therapies—such as anti-IFNα antibody (anifrolumab) and anti-IFNβ antibody (dazukibart)—across functional, transcriptional, and proteomic outcomes in muscle. Specifically, the myobundle model can be leveraged for preclinical drug screening, enabling the assessment of functional rescue through measurement of contractile force, inflammatory gene and protein signatures including muscle-related biomarkers, and drug-induced structural changes.

To our knowledge, this is the first study to show that IFN-stimulated human-derived muscle tissue can recapitulate inflammatory myokine production seen in JDM. Additional strengths of this study include reproducibility of cytokine effects across multiple pediatric donors from which myobundles were derived, correlation to a clinical cohort of treatment-naïve JDM patients in both blood and muscle samples with incorporation of healthy controls, and orthogonal validation of changes in myokines with both protein and transcriptomic approaches. Importantly, this study highlights the utility of myobundles as a platform to study cytokine-mediated pathology and test targeted interventions for JDM.

There are several limitations to this study. First, patient data was collected in a retrospective manner that precluded the formal use of diagnostic classification criteria (16–18), yet we note that patients’ features across sites were consistent with a diagnosis of JDM. Data was collected from an active disease, pre-treatment status only, which limited the scope of this study. Future studies could determine change in myokine profiles over time to investigate clinical correlation and response to specific therapeutic drugs. Second, the patient cohorts from which blood samples and muscle biopsies were collected were largely distinct, with only three overlapping JDM patients. Given the sample size and non-overlapping cohorts, direct comparisons could not be made across clinical, serological, and transcriptional datasets. The relatively small sample size and incomplete clinical data (e.g. PGA at the time of sample collection) limited statistical power and may have introduced some bias, given that the data were likely not missing at random. Additionally, there was high variability noted in patient data including traditional labs and myokine measurements, highlighting patient heterogeneity at disease presentation. Our study was intended to be exploratory and hypothesis generating, and we call for larger studies in the future to replicate and further clarify our findings. Finally, not all myokines measured were able to be orthogonally validated via RNA sequencing of muscle and myobundle samples due to low level expression of IFNα, IFNβ, IL-17A, and TNFα.

In conclusion, IFN-stimulated pediatric myobundles recapitulate key aspects of the inflammatory myokine profile seen in JDM. Divergence observed between patients and the model highlights the influence of systemic immunological cross-talk with muscle. We demonstrate that muscle may be a source of systemic and local tissue inflammation, driving disease activity. CXCL9 and CXCL10 show consistent promise as potential disease biomarkers, with IL-6, IL-15, IL-18, also warranting additional investigation as biomarkers of muscle inflammation. Importantly, our findings underscore the value of the myobundle model for investigating muscle-intrinsic disease mechanisms, clarifying systemic contributions of muscle to pathogenesis, and identifying potential biomarkers and therapeutic targets.

## Data Availability

The original contributions presented in the study are included in the article/**Supplementary Material**; further inquiries can be directed to the corresponding author.

## References

[1] Coss SL Sabbagh SE Kim H . Updates in juvenile dermatomyositis: pathogenesis and therapy. Curr Opin Rheumatol. (2025) 37:445–56. doi: 10.1097/BOR.0000000000001112, PMID: 40693918

[2] Moneta GM Pires Marafon D Marasco E Rosina S Verardo M Fiorillo C . Muscle expression of type I and type II interferons is increased in juvenile dermatomyositis and related to clinical and histologic features. Arthritis Rheumatol. (2019) 71:1011–21. doi: 10.1002/art.40800, PMID: 30552836

[3] Rodríguez-Carrio J Burska A Conaghan PG Dik WA Biesen R Eloranta ML . Association between type I interferon pathway activation and clinical outcomes in rheumatic and musculoskeletal diseases: a systematic literature review informing EULAR points to consider. RMD Open. (2023) 9. doi: 10.1136/rmdopen-2022-002864, PMID: 36882218 PMC10008483

[4] Pachman LM Nolan BE DeRanieri D Khojah AM . Juvenile dermatomyositis: new clues to diagnosis and therapy. Curr Treatm Opt Rheumatol. (2021) 7:39–62. doi: 10.1007/s40674-020-00168-5, PMID: 34354904 PMC8336914

[5] Neely J Ardalan K Huber A Kim S . Baseline characteristics of children with juvenile dermatomyositis enrolled in the first year of the new Childhood Arthritis and Rheumatology Research Alliance registry. Pediatr Rheumatol Online J. (2022) 20:50. doi: 10.1186/s12969-022-00709-3, PMID: 35854378 PMC9295519

[6] Seok J Warren HS Cuenca AG Mindrinos MN Baker HV Xu W . Genomic responses in mouse models poorly mimic human inflammatory diseases. Proc Natl Acad Sci U S A. (2013) 110:3507–12. doi: 10.1073/pnas.1222878110, PMID: 23401516 PMC3587220

[7] Afzali AM Ruck T Wiendl H Meuth SG . Animal models in idiopathic inflammatory myopathies: How to overcome a translational roadblock? Autoimmun Rev. (2017) 16:478–94. doi: 10.1016/j.autrev.2017.03.001, PMID: 28286105

[8] Covert LT Patel H Osman A Duncan L Dvergsten J Truskey GA . Effect of type I interferon on engineered pediatric skeletal muscle: a promising model for juvenile dermatomyositis. Rheumatol (Oxford). (2024) 63:209–17. doi: 10.1093/rheumatology/kead186, PMID: 37094222 PMC10765138

[9] Covert LT Prinz JA Swain-Lenz D Dvergsten J Truskey GA . Genetic changes from type I interferons and JAK inhibitors: clues to drivers of juvenile dermatomyositis. Rheumatol (Oxford). (2024) 63:Si240–si8. doi: 10.1093/rheumatology/keae082, PMID: 38317053 PMC11381683

[10] Mageriu V Manole E Bastian AE Staniceanu F . Role of myokines in myositis pathogenesis and their potential to be new therapeutic targets in idiopathic inflammatory myopathies. J Immunol Res. (2020) 2020:9079083. doi: 10.1155/2020/9079083, PMID: 32775472 PMC7396002

[11] Davis BNJ Santoso JW Walker MJ Oliver CE Cunningham MM Boehm CA . Modeling the effect of TNF-α upon drug-induced toxicity in human, tissue-engineered myobundles. Ann BioMed Eng. (2019) 47:1596–610. doi: 10.1007/s10439-019-02263-8, PMID: 30963383 PMC6559943

[12] Madden L Juhas M Kraus WE Truskey GA Bursac N . Bioengineered human myobundles mimic clinical responses of skeletal muscle to drugs. Elife. (2015) 4:e04885. doi: 10.7554/eLife.04885, PMID: 25575180 PMC4337710

[13] Greenberg SA . Type 1 interferons and myositis. Arthritis Res Ther. (2010) 12 Suppl 1:S4. doi: 10.1186/ar2885, PMID: 20392291 PMC2991777

[14] Baechler EC Bilgic H Reed AM . Type I interferon pathway in adult and juvenile dermatomyositis. Arthritis Res Ther. (2011) 13:249. doi: 10.1186/ar3531, PMID: 22192711 PMC3334651

[15] Zhan RZ Rao L Chen Z Strash N Bursac N . Loss of sarcomeric proteins via upregulation of JAK/STAT signaling underlies interferon-γ-induced contractile deficit in engineered human myocardium. Acta Biomater. (2021) 126:144–53. doi: 10.1016/j.actbio.2021.03.007, PMID: 33705988 PMC8096718

[16] Bohan A Peter JB . Polymyositis and dermatomyositis (first of two parts). N Engl J Med. (1975) 292:344–7. doi: 10.1056/NEJM197502132920706, PMID: 1090839

[17] Bohan A Peter JB . Polymyositis and dermatomyositis (second of two parts). N Engl J Med. (1975) 292:403–7. doi: 10.1056/NEJM197502202920807, PMID: 1089199

[18] Lundberg IE Tjärnlund A Bottai M Werth VP Pilkington C Visser M . 2017 European League Against Rheumatism/American College of Rheumatology classification criteria for adult and juvenile idiopathic inflammatory myopathies and their major subgroups. Ann Rheum Dis. (2017) 76:1955–64. doi: 10.1136/annrheumdis-2017-211468, PMID: 29079590 PMC5736307

[19] Chen S Zhou Y Chen Y Gu J . fastp: an ultra-fast all-in-one FASTQ preprocessor. Bioinformatics. (2018) 34:i884–i90. doi: 10.1093/bioinformatics/bty560, PMID: 30423086 PMC6129281

[20] Kersey PJ Staines DM Lawson D Kulesha E Derwent P Humphrey JC . Ensembl Genomes: an integrative resource for genome-scale data from non-vertebrate species. Nucleic Acids Res. (2012) 40:D91–7. doi: 10.1093/nar/gkr895, PMID: 22067447 PMC3245118

[21] Dobin A Davis CA Schlesinger F Drenkow J Zaleski C Jha S . STAR: ultrafast universal RNA-seq aligner. Bioinformatics. (2013) 29:15–21. doi: 10.1093/bioinformatics/bts635, PMID: 23104886 PMC3530905

[22] Love MI Huber W Anders S . Moderated estimation of fold change and dispersion for RNA-seq data with DESeq2. Genome Biol. (2014) 15:550. doi: 10.1186/s13059-014-0550-8, PMID: 25516281 PMC4302049

[23] Huber W Carey VJ Gentleman R Anders S Carlson M Carvalho BS . Orchestrating high-throughput genomic analysis with Bioconductor. Nat Methods. (2015) 12:115–21. doi: 10.1038/nmeth.3252, PMID: 25633503 PMC4509590

[24] Benjamini Y Hochberg Y . Controlling the false discovery rate: A practical and powerful approach to multiple testing. J R Stat Soc Ser B (Methodological). (1995) 57:289–300. doi: 10.1111/j.2517-6161.1995.tb02031.x

[25] Savan R . Post-transcriptional regulation of interferons and their signaling pathways. J Interferon Cytokine Res. (2014) 34:318–29. doi: 10.1089/jir.2013.0117, PMID: 24702117 PMC4015472

[26] Li MM MacDonald MR Rice CM . To translate, or not to translate: viral and host mRNA regulation by interferon-stimulated genes. Trends Cell Biol. (2015) 25:320–9. doi: 10.1016/j.tcb.2015.02.001, PMID: 25748385 PMC4441850

[27] Ivashkiv LB Donlin LT . Regulation of type I interferon responses. Nat Rev Immunol. (2014) 14:36–49. doi: 10.1038/nri3581, PMID: 24362405 PMC4084561

[28] Roberson EDO Mesa RA Morgan GA Cao L Marin W Pachman LM . Transcriptomes of peripheral blood mononuclear cells from juvenile dermatomyositis patients show elevated inflammation even when clinically inactive. Sci Rep. (2022) 12:275. doi: 10.1038/s41598-021-04302-8, PMID: 34997119 PMC8741808

[29] Ward JM Ambatipudi M O'Hanlon TP Smith MA de Los Reyes M Schiffenbauer A . Shared and distinctive transcriptomic and proteomic pathways in adult and juvenile dermatomyositis. Arthritis Rheumatol. (2023) 75:2014–26. doi: 10.1002/art.42615, PMID: 37229703 PMC10615891

[30] De Rossi M Bernasconi P Baggi F de Waal Malefyt R Mantegazza R . Cytokines and chemokines are both expressed by human myoblasts: possible relevance for the immune pathogenesis of muscle inflammation. Int Immunol. (2000) 12:1329–35. doi: 10.1093/intimm/12.9.1329, PMID: 10967028

[31] Nielsen AR Pedersen BK . The biological roles of exercise-induced cytokines: IL-6, IL-8, and IL-15. Appl Physiol Nutr Metab. (2007) 32:833–9. doi: 10.1139/H07-054, PMID: 18059606

[32] Podbregar M Lainscak M Prelovsek O Mars T . Cytokine response of cultured skeletal muscle cells stimulated with proinflammatory factors depends on differentiation stage. ScientificWorldJournal. (2013) 2013:617170. doi: 10.1155/2013/617170, PMID: 23509435 PMC3590685

[33] Wilson NJ Boniface K Chan JR McKenzie BS Blumenschein WM Mattson JD . Development, cytokine profile and function of human interleukin 17-producing helper T cells. Nat Immunol. (2007) 8:950–7. doi: 10.1038/ni1497, PMID: 17676044

[34] Akdis M Aab A Altunbulakli C Azkur K Costa RA Crameri R . Interleukins (from IL-1 to IL-38), interferons, transforming growth factor β, and TNF-α: Receptors, functions, and roles in diseases. J Allergy Clin Immunol. (2016) 138:984–1010. doi: 10.1016/j.jaci.2016.06.033, PMID: 27577879

[35] Fajgenbaum DC June CH . Cytokine storm. N Engl J Med. (2020) 383:2255–73. doi: 10.1056/NEJMra2026131, PMID: 33264547 PMC7727315

[36] Bellutti Enders F van Wijk F Scholman R Hofer M Prakken BJ van Royen-Kerkhof A . Correlation of CXCL10, tumor necrosis factor receptor type II, and galectin 9 with disease activity in juvenile dermatomyositis. Arthritis Rheumatol. (2014) 66:2281–9. doi: 10.1002/art.38676, PMID: 24756983

[37] Wienke J Pachman LM Morgan GA Yeo JG Amoruso MC Hans V . Endothelial and inflammation biomarker profiles at diagnosis reflecting clinical heterogeneity and serving as a prognostic tool for treatment response in two independent cohorts of patients with juvenile dermatomyositis. Arthritis Rheumatol. (2020) 72:1214–26. doi: 10.1002/art.41236, PMID: 32103637 PMC7329617

[38] Oda K Kotani T Takeuchi T Ishida T Shoda T Isoda K . Chemokine profiles of interstitial pneumonia in patients with dermatomyositis: a case control study. Sci Rep. (2017) 7:1635. doi: 10.1038/s41598-017-01685-5, PMID: 28487565 PMC5431618

[39] Turnier JL Pachman LM Lowe L Tsoi LC Elhaj S Menon R . Comparison of lesional juvenile myositis and lupus skin reveals overlapping yet unique disease pathophysiology. Arthritis Rheumatol. (2021) 73:1062–72. doi: 10.1002/art.41615, PMID: 33305541 PMC8274390

[40] Lv J Li L Li W Ji K Hou Y Yan C . Role of the chemokine receptors CXCR3, CXCR4 and CCR7 in the intramuscular recruitment of plasmacytoid dendritic cells in dermatomyositis. J Neuroimmunol. (2018) 319:142–8. doi: 10.1016/j.jneuroim.2018.01.008, PMID: 29366593

[41] Wienke J Bellutti Enders F Lim J Mertens JS van den Hoogen LL Wijngaarde CA . Galectin-9 and CXCL10 as biomarkers for disease activity in juvenile dermatomyositis: A longitudinal cohort study and multicohort validation. Arthritis Rheumatol. (2019) 71:1377–90. doi: 10.1002/art.40881, PMID: 30861625 PMC6973145

[42] Loaiza-Félix J Moreno-Ramírez M Pérez-García FL Jiménez-Rojas V Sánchez-Muñoz F Amezcua-Guerra ML . Serum levels of adipokines in patients with idiopathic inflammatory myopathies: a pilot study. Rheumatol Int. (2017) 37:1341–5. doi: 10.1007/s00296-017-3752-z, PMID: 28536758

[43] Sugiura T Kawaguchi Y Harigai M Takagi K Ohta S Fukasawa C . Increased CD40 expression on muscle cells of polymyositis and dermatomyositis: role of CD40-CD40 ligand interaction in IL-6, IL-8, IL-15, and monocyte chemoattractant protein-1 production. J Immunol. (2000) 164:6593–600. doi: 10.4049/jimmunol.164.12.6593, PMID: 10843719

[44] Yang Y Yin G Hao J Xie Q Liu Y . Serum interleukin-18 level is associated with disease activity and interstitial lung disease in patients with dermatomyositis. Arch Rheumatol. (2017) 32:181–8. doi: 10.5606/ArchRheumatol.2017.6175, PMID: 30375533 PMC6190947

[45] Das DK Graham ZA Cardozo CP . Myokines in skeletal muscle physiology and metabolism: Recent advances and future perspectives. Acta Physiol (Oxf. (2020) 228:e13367. doi: 10.1111/apha.13367, PMID: 31442362

[46] Nadeau L Patten DA Caron A Garneau L Pinault-Masson E Foretz M . IL-15 improves skeletal muscle oxidative metabolism and glucose uptake in association with increased respiratory chain supercomplex formation and AMPK pathway activation. Biochim Biophys Acta Gen Subj. (2019) 1863:395–407. doi: 10.1016/j.bbagen.2018.10.021, PMID: 30448294 PMC6310627

[47] Bilgic H Ytterberg SR Amin S McNallan KT Wilson JC Koeuth T . Interleukin-6 and type I interferon-regulated genes and chemokines mark disease activity in dermatomyositis. Arthritis Rheumatol. (2009) 60:3436–46. doi: 10.1002/art.24936, PMID: 19877033

[48] Neely J Hartoularos G Bunis D Sun Y Lee D Kim S . Multi-modal single-cell sequencing identifies cellular immunophenotypes associated with juvenile dermatomyositis disease activity. Front Immunol. (2022) 13:902232. doi: 10.3389/fimmu.2022.902232, PMID: 35799782 PMC9254730

[49] Miossec P Korn T Kuchroo VK . Interleukin-17 and type 17 helper T cells. N Engl J Med. (2009) 361:888–98. doi: 10.1056/NEJMra0707449, PMID: 19710487

[50] Tucci M Quatraro C Dammacco F Silvestris F . Interleukin-18 overexpression as a hallmark of the activity of autoimmune inflammatory myopathies. Clin Exp Immunol. (2006) 146:21–31. doi: 10.1111/j.1365-2249.2006.03180.x, PMID: 16968394 PMC1809738

[51] Helmers SB Bruton M Loell I Ulfgren AK Gracie AJ McInnes IB . Expression of interleukin-18 in muscle tissue of patients with polymyositis or dermatomyositis and effects of conventional immunosuppressive treatment. Rheumatol (Oxford). (2018) 57:2149–57. doi: 10.1093/rheumatology/key222, PMID: 30102381

[52] Schall TJ Bacon K Toy KJ Goeddel DV . Selective attraction of monocytes and T lymphocytes of the memory phenotype by cytokine RANTES. Nature. (1990) 347:669–71. doi: 10.1038/347669a0, PMID: 1699135

[53] Lightfoot AP Goljanek-Whysall K Cotton CV Earl KE McArdle A Cooper RG . O42. Is muscle a chemotactic organ in the idiopathic inflammatory myopathies (IIM)? Overexpression of MHC I (H-2Kb) in C2C12 myotubes results in release of pro-inflammatory cytokines. Rheumatology. (2015) 54:i43–i. doi: 10.1093/rheumatology/kev085.006

[54] Civatte M Bartoli C Schleinitz N Chetaille B Pellissier JF Figarella-Branger D . Expression of the beta chemokines CCL3, CCL4, CCL5 and their receptors in idiopathic inflammatory myopathies. Neuropathol Appl Neurobiol. (2005) 31:70–9. doi: 10.1111/j.1365-2990.2004.00591.x, PMID: 15634233

[55] De Bleecker JL De Paepe B Vanwalleghem IE Schröder JM . Differential expression of chemokines in inflammatory myopathies. Neurology. (2002) 58:1779–85. doi: 10.1212/WNL.58.12.1779, PMID: 12084877

[56] Moran EM Mastaglia FL . The role of interleukin-17 in immune-mediated inflammatory myopathies and possible therapeutic implications. Neuromuscul Disord. (2014) 24:943–52. doi: 10.1016/j.nmd.2014.06.432, PMID: 25052503

[57] Silva MG Oba-Shinjo SM Marie SKN Shinjo SK . Serum interleukin-17A level is associated with disease activity of adult patients with dermatomyositis and polymyositis. Clin Exp Rheumatol. (2019) 37:656–62., PMID: 30620283

[58] Higgs BW Zhu W Richman L Fiorentino DF Greenberg SA Jallal B . Identification of activated cytokine pathways in the blood of systemic lupus erythematosus, myositis, rheumatoid arthritis, and scleroderma patients. Int J Rheum Dis. (2012) 15:25–35. doi: 10.1111/j.1756-185X.2011.01654.x, PMID: 22324944

[59] Chevrel G Page G Granet C Streichenberger N Varennes A Miossec P . Interleukin-17 increases the effects of IL-1 beta on muscle cells: arguments for the role of T cells in the pathogenesis of myositis. J Neuroimmunol. (2003) 137:125–33. doi: 10.1016/S0165-5728(03)00032-8, PMID: 12667656

[60] Kocić J Santibañez JF Krstić A Mojsilović S Dorđević IO Trivanović D . Interleukin 17 inhibits myogenic and promotes osteogenic differentiation of C2C12 myoblasts by activating ERK1,2. Biochim Biophys Acta. (2012) 1823:838–49. doi: 10.1016/j.bbamcr.2012.01.001, PMID: 22285818

[61] Sag E Kale G Haliloglu G Bilginer Y Akcoren Z Orhan D . Inflammatory milieu of muscle biopsies in juvenile dermatomyositis. Rheumatol Int. (2021) 41:77–85. doi: 10.1007/s00296-020-04735-w, PMID: 33106894

[62] Zou J Kawai T Tsuchida T Kozaki T Tanaka H Shin KS . Poly IC triggers a cathepsin D- and IPS-1-dependent pathway to enhance cytokine production and mediate dendritic cell necroptosis. Immunity. (2013) 38:717–28. doi: 10.1016/j.immuni.2012.12.007, PMID: 23601685

[63] Dempoya J Matsumiya T Imaizumi T Hayakari R Xing F Yoshida H . Double-stranded RNA induces biphasic STAT1 phosphorylation by both type I interferon (IFN)-dependent and type I IFN-independent pathways. J Virol. (2012) 86:12760–9. doi: 10.1128/JVI.01881-12, PMID: 22973045 PMC3497619

