# Supplementary figures and images for: Unmasking inflammation in juvenile dermatomyositis: myokine profiles of patients and bioengineered human muscle
Source: Front Immunol. 2025 Dec 18;16:1694717. doi: 10.3389/fimmu.2025.1694717 (PMC12756377; doi:10.3389/fimmu.2025.1694717)

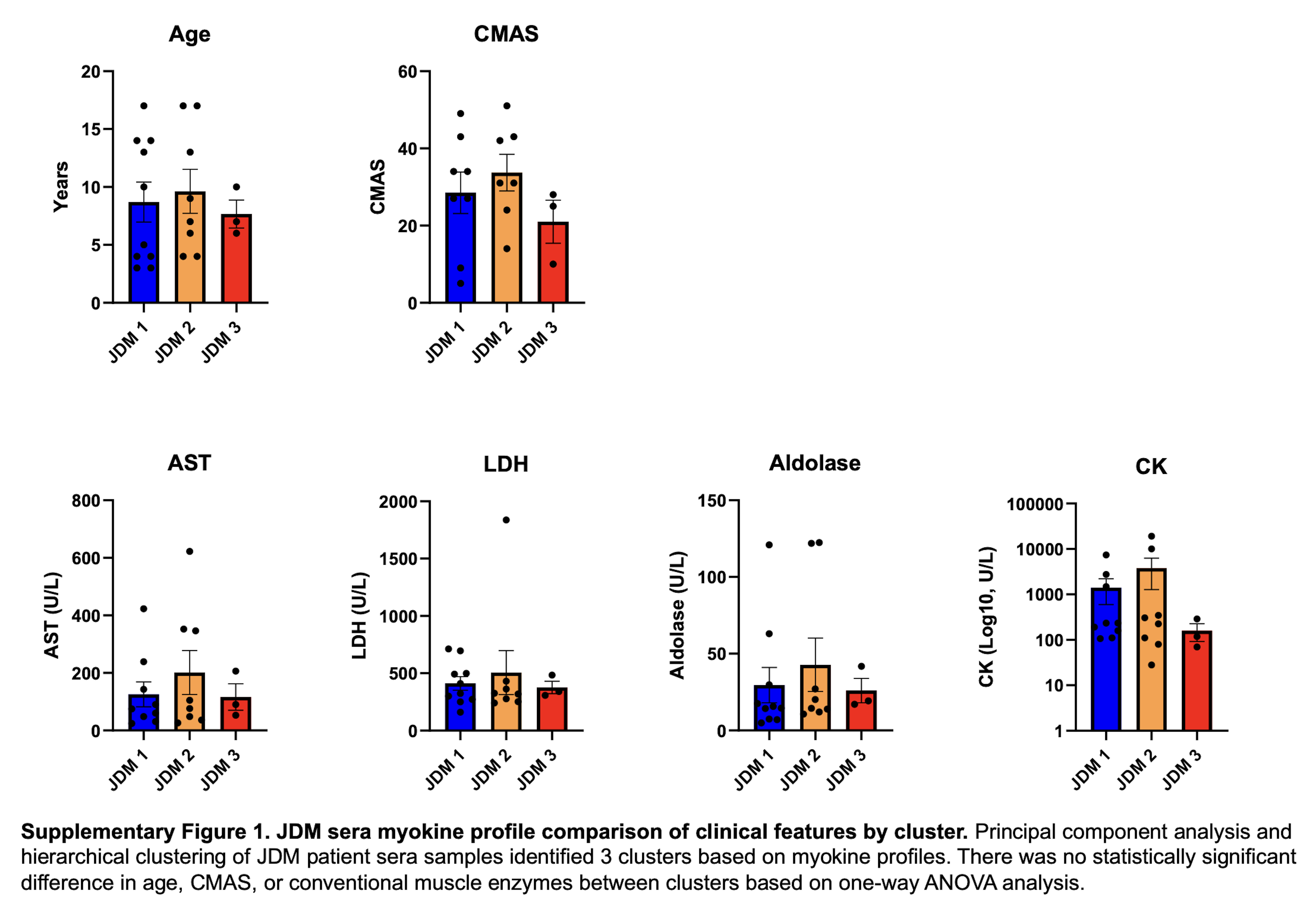

Supplement: Supplementary file 1 [file Image1.jpeg]

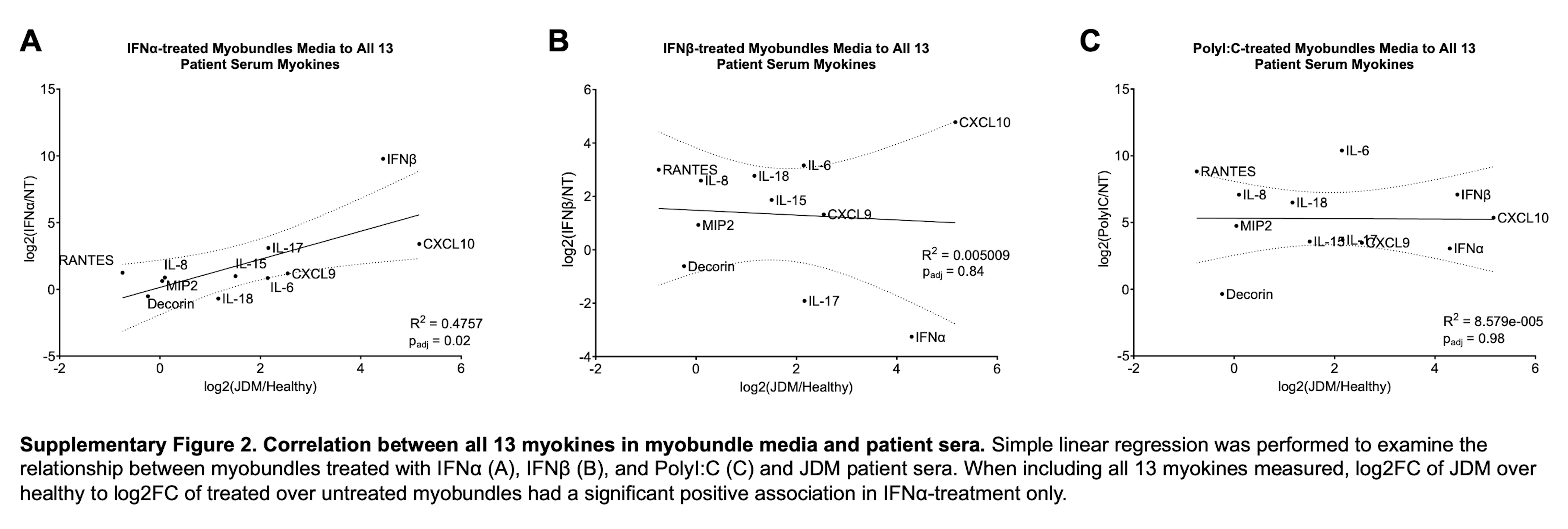

Supplement: Supplementary file 2 [file Image2.jpeg]
