# Supplementary material for: Unmasking inflammation in juvenile dermatomyositis: myokine profiles of patients and bioengineered human muscle
Source: Front Immunol. 2025 Dec 18;16:1694717. doi: 10.3389/fimmu.2025.1694717 (PMC12756377; doi:10.3389/fimmu.2025.1694717)
Supplement: Supplementary file 3 [file DataSheet1.docx]

**Supplementary Text S1. Description of JDM patient serum cohort**

Mean age of the JDM patients was 8.9 (+ 1.1) years with 81% being female. Majority (52.4%) were non-Hispanic white, followed by Hispanic (23.8%), and black patients (19%). Average Childhood Myositis Assessment Scale (CMAS) at time of serum collection was 29.3 (+ 3.2) out of 52 maximum points, indicating moderate to severe muscle disease activity.^17^ Mean Physician’s Global Assessment (PGA) was 5.4 (SD 0.6), with scores of 0 indicating no disease activity and scores of 10 indicating very severe disease activity. It is noted that 57.1% of the patient cohort did not have a PGA assigned at the time of sera collection. 61.9% of patients had a myositis-specific antibody (MSA) present, with 19% of these MSA positive for anti-MJ/NXP2. Of note, the patient with positive PL-12 antibody was only weakly positive (Mayo Myomarker 3 panel) and did not have typical manifestations of associated anti-synthetase syndrome. Rather, this patient was diagnosed with and treated for JDM. 23.8% of patients were positive for a myositis-associated antibody (MAA). Of the two patients testing positive for RNP antibody, one was weakly positive for U1RNP (Oklahoma Medical Research Foundation panel) and did not have features of overlap myositis or mixed connective tissue disease, while the other +RNP patient had high-titer U2RNP and negative U1RNP (Mayo). While this patient had Raynaud phenomenon, they did not have other features of overlap myositis or scleroderma-spectrum disease and were ultimately diagnosed with and treated clinically for JDM. Finally, the patient who had positive PM-SCL was only weakly positive (Mayo Myomarker 3 panel); they did not have features of overlap myositis and were also diagnosed and treated for JDM. Thus, all patients listed in Supp. Table 1 were included for this study as they met all inclusion criteria with all clinical phenotypes consistent with JDM.
